# An expert curated global legume checklist improves the accuracy of occurrence, biodiversity and taxonomic data

**DOI:** 10.1038/s41597-022-01812-6

**Published:** 2022-11-17

**Authors:** M. Marianne le Roux, Joseph T. Miller, John Waller, Markus Döring, Anne Bruneau

**Affiliations:** 1 Foundational Biodiversity Sciences Division, South African National Biodiversity Institute, Pretoria, South Africa; 2 Department of Botany and Plant Biotechnology, University of Johannesburg, Johannesburg, South Africa; 3 Global Biodiversity Information Facility, Copenhagen, Denmark; 4 Institut de Recherche en Biologie Végétale & Département de Sciences Biologiques, Université de Montréal, Montréal, Canada

**Keywords:** Plant sciences, Databases

## Abstract

The Legume Phylogeny Working Group’s Taxonomy Working Group was tasked to create a community endorsed global legume checklist that will serve as a primary source of taxa for biodiversity data platforms and legume-related research. The checklist was published in June 2021, recognising 772 genera and 22,360 species. It is disseminated through the new Legume Data Portal as part of the Global Biodiversity Information Facility (GBIF) hosted portal initiative. The process that was followed to publish and disseminate the checklist and its content is described here. The impact of the work by the Taxonomy Working Group are quantified by comparing the published checklist with the GBIF taxonomic backbone. A total of 44,157 names overlapped with the GBIF taxonomic backbone while 30,456 names were added, which enabled more accurate name matching of 61,235 legume occurrences. Continuous improvement to the World Checklist of Vascular Plants (WCVP): Fabaceae checklist will allow the GBIF taxonomic backbone and other checklist managers to converge to a consistent and comprehensive list of legume taxa globally over time.

## Abstract (summary)

**Table:** 

Measurement(s)	Corrections and impact
Technology Type(s)	Data comparisons
Factor Type(s)	taxon name
Sample Characteristic - Organism	Fabaceae
Sample Characteristic - Environment	global

## Background & Summary

Scientific names are central to biodiversity communication and applied research because they link all types of data to make related information about species discoverable. Species checklists capture names of organisms that are organised into higher levels of classification, e.g. genera, tribes and families. Many online resources are available to keep track of taxa in the form of checklists, for example the African Plant Database (http://africanplantdatabase.ch), Catalogue of Life (COL, https://www.catalogueoflife.org), World Checklist of Vascular Plants (WCVP)^1^ and World Flora Online^2^ Plant List (https://wfoplantlist.org/plant-list) while other platforms such as the Global Biodiversity Information Facility (GBIF, https://www.gbif.org) and GenBank (https://www.ncbi.nlm.nih.gov/genbank) use taxa from these checklists to index and share linked biodiversity data. However, keeping abreast the continuous changes in taxonomy as a result of an improved understanding of species limits and relationships using molecular and evolutionary methods and the discovery of new species may be difficult. Accurate record-keeping of accepted names and their synonyms is critical but challenging and relies heavily on input, often from the same small pool of taxon experts.

**Fig. 1:**
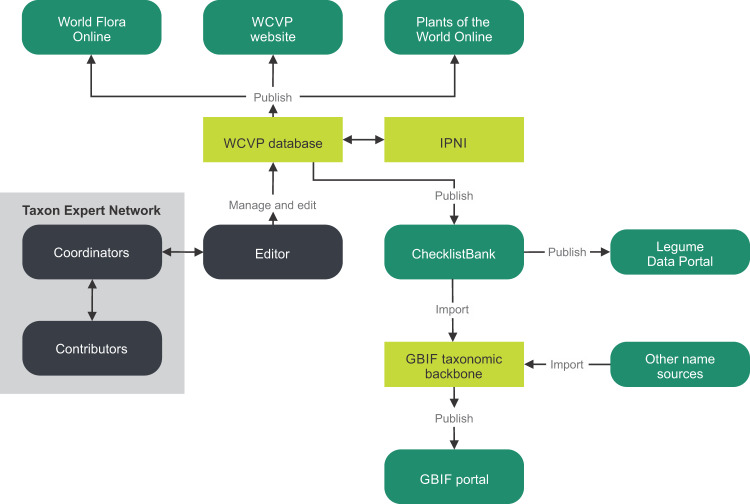
An overview of the workflow that was followed to update and publish the World Checklist of Vascular Plants (WCVP): Fabaceae checklist. Contributors, coordinators and the editor communicated back and forth to update legume taxa in the WCVP database. Content in the WCVP database were cross-referenced with the International Plant Name Index (IPNI). Data within the WCVP database were disseminated through various online platforms. The legume component of the WCVP database was published as a Darwin Core Archive in ChecklistBank from where it was integrated into the Global Biodiversity Information Facility (GBIF) taxonomic backbone and published online through ChecklistBank (https://www.checklistbank.org/dataset/2304/about) on the Legume Data Portal (https://www.legumedata.org) and the GBIF portal^6^.

The legume systematics community has a strong history of collaboration to increase understanding of Fabaceae (or Leguminosae, Article 18.5^3^) evolution and to improve the classification system. This is evident in the International Legume Conferences held since 1978, the publication of over 14 volumes of the series Advances in Legume Systematics^4^, the establishment of the International Legume Database and Information Service (ILDIS, https://www.ildis.org)^4,5^ and of the Legume Phylogeny Working Group (LPWG)^4^. The International Legume Database and Information Service (ILDIS) was a leading example globally of a taxon specific online resource that contained a checklist with full synonymy, categorical data on geographic distributions and life-history traits^5^ but since the early 2000s, limited work has been done on maintaining and expanding the database. It was highlighted that a new online species-information system is needed^4^, an idea confirmed at a LPWG meeting held in May 2020 (https://www.legumedata.org/beanbag/67/67content). As a result, the LPWG Taxonomy Working Group was established to create a community endorsed consensus global checklist that can serve as a primary source of legume scientific taxa to support research (including work planned on phylogenomics, occurrences, traits and a contribution towards the Open Tree of Life^4^) and to benefit online resources that link data using names and subsequently taxa (Fig. 1).

A collaboration was set up between the LPWG Taxonomy Working Group and the Royal Botanic Gardens, Kew, to review and update the existing legume component of the World Checklist of Vascular Plants (WCVP). The first updated WCVP legume checklist (vers. 2021; hereafter referred to as the WCVP: Fabaceae checklist) was edited by an international cohort of legume taxonomists and published on ChecklistBank (https://www.checklistbank.org/dataset/2304/about), an open collaborative checklist repository and taxonomy resource led by GBIF and COL.

In 2021 GBIF initiated a hosted portal pilot program for showcasing occurrence data of specific scientific communities or regions. In collaboration with Canadensys (www.canadensys.net), a GBIF Associate Participant Node, a legume data thematic portal was created and is reported for the first time here. The main component of this GBIF hosted portal is a view of Fabaceae GBIF occurrence (observation and specimen) data. The LPWG used this opportunity to showcase legume information (https://www.legumedata.org), including sharing the WCVP: Fabaceae checklist^6,7^ and to develop a community resource. Concurrently, this gave GBIF an opportunity to measure the community needs and increase GBIF data quality by facilitating automatic taxon updates to improve the GBIF taxonomic backbone^8^ as well as the taxonomy of its collaborator, COL and other international initiatives like World Flora Online^7^ for which the LPWG is considered the recognised Taxon Expert Network^2^.

This paper is aimed to (1) explain the workflow that was followed to update and publish the community endorsed legume checklist, (2) to define the checklist’s content, (3) to outline data dissemination of the checklist on the new Legume Data Portal and other portals, and (4) to describe the integration of the legume checklist into the GBIF taxonomic backbone and consequently measure improvements of occurrence data in GBIF to illustrate the importance of taxon expert curation of checklists.

## Methods

An explanation of the steps that were followed to set up the taxonomist network that curated the checklist is provided along with the data flow and publication of the checklist. A detailed description is provided outlining the data content of the first published community endorsed consensus legume checklist^7^ along with an evaluation of the impact of checklist improvements on biodiversity data using GBIF occurrence data as a case study.

### Starting list of taxa and updates

The LPWG Taxonomy Working Group had to find the most up-to-date checklist to use as a starting point in generating a global community endorsed checklist. The WCVP is actively maintained by the Royal Botanic Gardens, Kew in four steps^9^: (1) Recording of accepted names and synonyms (A-Z workflow), (2) endorsement of the list by a taxon expert community, (3) recording of geographic distributions, and 4) maintaining the checklist by updating with new names when they are validly published. Since this is an actively curated checklist, it was decided to use the WCVP list as the starting list by collaborating with the Royal Botanic Gardens, Kew and maintaining the legume checklist within this environment. The LPWG Taxonomy Working Group and the Royal Botanic Gardens, Kew joined forces by connecting at step 2. This list has the added benefit of regular cross-checks with the International Plant Name Index (https://www.ipni.org) and an editor (Fig. 1) with an in-depth knowledge of the scientific naming rules^3^. The start list included a total of 85,811 taxa (Supplementary Table 1) and updates were made based on published literature, taxonomic expertise, and, as needed, the study of type specimens (physical specimens, images of type material on e.g. Global Plants on JSTOR, https://plants.jstor.org). Taxonomic statuses used included accepted, artificial hybrid, illegitimate, incomplete, invalid, misapplied, orthographic variant, synonym or unplaced. The term ‘unplaced’ refers to any taxonomic status, other than ‘accepted’, that could not be confirmed or verified by an expert. In the case of conflicting taxonomies that existed simultaneously, the coordinator mediated between the respective groups to engage with one another to reach consensus through discussion and consultation. Although no case was referred to the arbitration committee for decision, such a committee exists (elected by the community and with members representing all major groups within the family and across all continents) to evaluate the case by (1) determining if the implementation of a particular taxonomy will create taxonomic instability, (2) attempt achieving philosophical consistency when defining lineages, or (3) by voting as a last resort.

### Network of legume taxonomists and the data

The LPWG Taxonomy Working Group created a network of coordinators and contributors (taxon experts) to check and verify taxa. In total, 37 coordinators [at least one for each subfamily, and for each tribe in the Papilionoideae subfamily] were tasked with finding experts to participate. The checklist along with instructions were circulated via the LPWG network to taxon experts who then volunteered to assist with taxon verification, starting in July 2020. A total of 80 taxonomists from 24 countries participated in the process^6,7^.

The legume component of the WCVP was prepared in .xlsx format and included 19 fields of data or associated fields that were used to sort the synonyms under their respective accepted names (Table 1). A ‘comments’ field was used to record corrections (and comments) made by the contributors and the ‘verified’ field to add the name of the taxon expert who checked and corrected or verified the data.

**Table 1:** Definitions for each field in the starting list of legume names extracted from the World Checklist of Vascular Plants.

Field name	Definition/Purpose
sort_name	Identifier associated with the accepted_name
plant_name_id	World Checklist of Vascular Plants identifier
ipni_id	International Plant Name Index identifier
family	Family name associated with the taxon_name
subfamily	Subfamily name associated with the accepted_name
tribe/clade	Tribe/clade name associated with the accepted_name
rank	The level within the hierarchical ranking system where the taxon belongs
taxon_status	The status of the use of the taxon_name, based on taxonomic opinion as a label for a taxon
taxon_name	The genus name, specific epithet and, where applicable, infraspecific epithet to form a monomial, binomial or trinomial name with its authorship
place_of_publication	The protologue citation of the taxon_name
volume_and_page	The protologue volume (where applicable) and page number of the taxon_name
first_published	The protologue year of publication of the taxon_name
geographic_area	Geographic coverage of the taxon_name
nomenclatural_remarks	Nomenclatural notes related to the taxon_name
accepted_plant_name_id	World Checklist of Vascular Plants identifier associated with the accepted_name
acc_ipni_id	The International Plant Name Index identifier associated with the accepted_name
accepted_name	The genus name, specific epithet and, where applicable, infraspecific epithet associated with the accepted_name to form a monomial, binomial or trinomial name with its authorship
comments	Corrections or comments recorded by the contributor
verified	Name of the contributor who corrected data or verified that the data for the record are accurate and correct

The dataset was checked, corrections noted in the ‘comments’ field and the name of the contributor(s)/taxon expert(s) who updated or verified the details of the particular record captured in the ‘verified’ field. A complete list of taxa is available in Supplementary Table 1.

The multiple .xlsx files with taxon verifications and corrections were collated manually by the coordinators, consolidated where multiple contributors worked on the same group, and submitted to the WCVP editor at the Royal Botanic Gardens, Kew who then conducted a comprehensive nomenclature assessment, following the International Code of Nomenclature^3,9^, before incorporating updates into the WCVP database. At the end of May 2021, all corrections were incorporated and the new list was reviewed by the community to publish a first updated checklist in June 2021.

### Workflow of data publication

Once corrections in the WCVP database were captured, the legume component was extracted and published as the WCVP: Fabaceae checklist in a Darwin Core Archive on ChecklistBank (https://www.checklistbank.org/dataset/2304/about, Table 2) and integrated into the GBIF taxonomic backbone^10^. The WCVP: Fabaceae checklist was used as the source dataset in the Legume Data Portal (https://www.legumedata.org) and as the priority dataset in the GBIF taxonomic backbone for the family. Consequently, since June 2021, all GBIF verbatim names of occurrences that form part of Fabaceae are indexed using the taxonomic interpretation of the LPWG Taxonomy Working Group^10,11^. However, GBIF also receives many names from other sources, e.g. Plazi (https://plazi.org), International Union for Conservation of Nature (https://www.iucn.org). Therefore, even though using the WCVP: Fabaceae checklist as its core dataset, the GBIF taxonomic backbone is augmented by other taxonomic datasets that add names currently not covered by the WCVP: Fabaceae checklist.

**Table 2:** The field index numbers, their related Darwin Core terms and definitions that define the fields in the WCVP: Fabaceae checklist.

Field index	DwC term	Note
0	taxonID	World Checklist of Vascular Plants identifier
1	family	The full scientific name of the family in which the scientificName is classified
2	genus	The full scientific name of the genus in which the scientificName belongs
3	specificEpithet	The name of the first or species epithet of the scientificName
4	infraSpecificEpithet	The name of the lowest or terminal infraspecific epithet of the scientificName, excluding any rank designation
5	scientificName	The genus name, where applicable, associated with the specificEpithet and infraspecific epithet to form a monomial, binomial or trinomial name without its authorship
6	scientificNameAuthorship	The authorship information for the scientificName formatted according to a standard convention^3^
7	taxonRank	The taxonomic rank of the most specific name in the scientificName
8	taxonomicStatus	The status of the use of the scientificName based on taxonomic opinion, linked to a specific taxonomic reference that defines the concept
9	acceptedNameUsageID	World Checklist of Vascular Plants identifier of the associated accepted name
10	parentNameUsageID	World Checklist of Vascular Plants identifier of the associated name one level up in the taxonomic hierarchy
11	originalNameUsageID	World Checklist of Vascular Plants identifier of the associated basionym
12	namePublishedIn	A reference for the publication (protologue citation) in which the scientificName was originally established^3^
13	scientificNameID	International Plant Name Index identifier
14	wfoID	World Flora Online identifier

The checklist is available for download as a Darwin Core Archive through redirection from ChecklistBank’s About page (https://www.checklistbank.org/dataset/2304/about), Zenodo^7^ or the Legume Data Portal (https://www.legumedata.org).

Data in the WCVP database are continuously made available online through the WCVP website (https://wcvp.science.kew.org) where individual records can be searched or where the entire dataset can be downloaded as the WCVP quarterly download (a link which allows one to download the complete checklist stored in the WCVP database) and the Plants of the World Online portal (https://powo.science.kew.org)^9^.

The LPWG Taxonomy Working Group is recognised as the Taxon Expert Network of legumes for the World Flora Online. The WCVP: Fabaceae checklist is also shared with the World Flora Online for incorporation into the World Flora Online Taxonomic Backbone^12^ which will take place after every major update to the WCVP: Fabaceae checklist (initially on an annual basis). As the Taxon Expert Network for legumes, the LPWG Taxonomy Working Group will also be responsible to resolve queries related to taxa in the World Flora Online Taxonomic Backbone, which in turn will help to make the WCVP: Fabaceae checklist comprehensive.

### GBIF Hosted portals: the legume data portal

The Legume Data Portal provides up to date taxonomic information resulting from work done by the LPWG Taxonomy Working Group. It also includes descriptive information for the family and subfamilies, occurrence data, background information about the working groups within the LPWG, access to The Bean Bag (a community newsletter) and other news. The WCVP: Fabaceae checklist, published on ChecklistBank, is visualised in the Taxonomy page using a JS React component library from Catalogue of Life (https://github.com/CatalogueOfLife/portal-components). This widget shows all data, including synonyms, present in the WCVP: Fabaceae checklist and allows substantial browse and search capabilities. Future updates to the WCVP: Fabaceae checklist data will be deposited to ChecklistBank and the widget can be directed to the updated taxonomy.

One of the main features of the hosted portal is the occurrence search of the entire GBIF Fabaceae dataset. The majority of GBIF occurrences are indexed based on the WCVP: Fabaceae checklist; however, as noted earlier, there are occurrences that match taxonomic names not in the WCVP: Fabaceae checklist but from other taxonomic resources. Therefore the taxonomy of the occurrences in the Legume Data Portal is not identical to the taxonomy presented in the Taxonomic page of the Legume Data Portal. It is expected that further rounds of expert curation will lessen these differences because the WCVP: Fabaceae checklist will continually grow to become more comprehensive. The goal is for each taxon to be assigned a single accepted name with the nomenclatural statuses of all synonyms flagged accordingly^12^. The increasingly comprehensive checklist will over time enable more accurate indexing and linking of taxa associated with GBIF occurrences.

### Data improvement evaluation

**Fig. 2:**
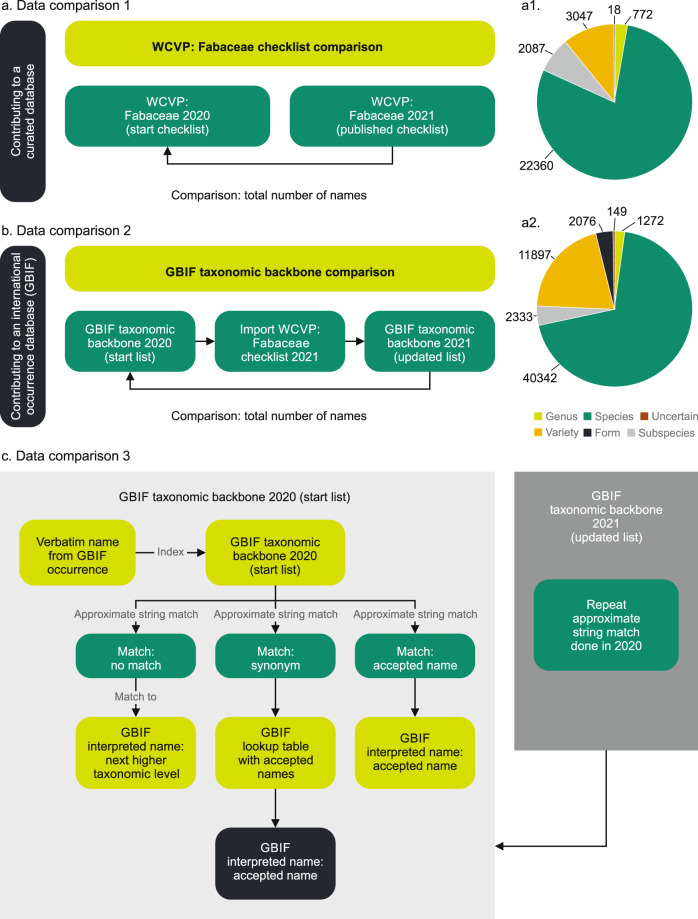
Three comparisons made with data from the published WCVP: Fabaceae checklist. a. A comparison between the starting checklist from 2020 and the WCVP: Fabaceae checklist^7^ (https://www.checklistbank.org/dataset/2304/about) published in June 2021 to determine the number of new names to the dataset recorded. a1. A pie chart showing the composition by rank of accepted taxa in the published checklist. a2. A pie chart showing the composition by rank of synonyms in the published checklist. b. A comparison of the GBIF taxonomic backbone before^13^ and after^10^ the incorporation of the WCVP: Fabaceae checklist. c. A comparison of GBIF occurrence name matches (verbatim names vs GBIF interpreted names) before^14^ and after^11^ incorporating the WCVP: Fabaceae checklist into the GBIF taxonomic backbone.

To determine the impact of the work done by the LPWG Taxonomy Working Group, three data comparisons were made (Fig. 2). Firstly, a comparison was made between the starting checklist (Supplementary Table 1) and the WCVP: Fabaceae 2021 checklist^7^. ChecklistBank metrics were used to calculate the total number of records for accepted genera, species, subspecies, varieties, forms, and synonyms and is available on GitHub (https://github.com/jhnwllr/The-World-Checklist-of-Vascular-Plants–Fabaceae). These values were compared between the start checklist and the published checklist to determine the total number of differences in names recorded by the LPWG Taxonomy Working Group.

Secondly, the WCVP: Fabaceae^7^ checklist was compared with the GBIF taxonomic backbone^10,13^. This allowed quantification of three values: (1) the number of new names introduced from the WCVP: Fabaceae checklist^7^ into the GBIF taxonomic backbone^10,13^, (2) the names in common between the WCVP: Fabaceae checklist and the GBIF taxonomic backbone, and (3) the number of names in the GBIF taxonomic backbone not included in the WCVP: Fabaceae checklist.

Thirdly, an analysis was done to quantify the effect of the taxonomic updates on occurrence data^11,14^ in GBIF as a result of the improvements in the GBIF taxonomic backbone^10^ with the WCVP: Fabaceae checklist^7^. Each GBIF occurrence (specimen or observation) arrives in GBIF with a taxonomic name, the verbatim (source/original) name. During the indexing process the verbatim name is matched to the GBIF taxonomic backbone. In the best case scenario, the verbatim name matches an accepted name in the GBIF taxonomic backbone and is then used as the GBIF interpreted name. If the verbatim name matches a synonym in the GBIF taxonomic backbone, the accepted name is derived from a lookup table and is used as the GBIF interpreted name (Fig. 2). The verbatim name is maintained.

In some cases, even with approximate string matching, there is no match of the verbatim name to an interpreted name in the GBIF taxonomic backbone. In this case, the algorithm used in the building of the index identifies the name to the next higher taxonomic level. For example if an occurrence has a verbatim name of *Acacia xyz*, which is not recognized in the GBIF taxonomic backbone, that occurrence is given the interpreted name of *Acacia* and no species name is inferred. Likewise occurrences with unmatched generic names would identify the record to family level and unmatched infraspecific names (e.g. subspecies, varieties, forms) would identify the record to species level. Similarly if a verbatim name has more than one potential equally best interpreted matches, it identifies the next higher level taxonomic name.

## Data Records

### Legume checklist data

The published version of the checklist includes a total number of 87,822 names and is accessible through ChecklistBank (https://www.checklistbank.org/dataset/2304/about), the GBIF website^6^ (https://www.gbif.org), Zenodo^7^ and Legume Data Portal (https://www.legumedata.org). The complete checklist is available as a Darwin Core Archive that consists of three files: 1) An EML metadata file^15^ (eml.xml), 2) a DwC archive descriptor file (meta.xml) and, 3) a taxon file with all taxa (wcvpFabaceae_June2021_withWFOIDs.txt). The field names (Darwin Core terms) in the taxon file are provided in the meta.xml file and are listed in Table 2.

## Technical Validation

The LPWG Taxonomy Working Group decided that taxon experts (contributors) would evaluate checklist data for their taxon groups of speciality. Corrections in the checklist are implemented based on published literature that is supported by sound evidence, often the work of these taxon experts themselves. When contentious names arose, they were addressed in collaboration with other experts to ensure that decisions were taken in a consistent manner within the context of the taxon group in question. Global collaboration is critical in maintaining a community endorsed checklist and therefore connections were established to link taxon experts that were not yet working together. An arbitration committee was set up in case conflicts needed to be evaluated by an objective group of experts, but this committee has yet to intervene in taxonomic decisions.

Corrections were submitted to the WCVP editor who performed a quality assessment of all the proposed updates^3,9^ before incorporation into the WCVP database. The editor could correspond with the contributors where questions arose before changes were made in the database. Once all corrections were recorded, a checklist was made available to the contributors for review before it was finally published.

In the first round of updating the legume checklist, not all taxonomic groups were addressed. Table 3 provides a summary of the total number of genera (excluding unplaced genera) recognised within each subfamily along with the respective percentages of genera evaluated to before June 2021.

**Table 3:** The percentage of genera (excluding unplaced genera) within each subfamily of the Fabaceae that have been verified in the WCVP: Fabaceae checklist in its first round of evaluation by the legume contributors (taxon experts).

Subfamily	Number of genera	% of genera verified
Caesalpinioideae	149	3
Cercidoideae	14	100
Detarioideae	76	87
Dialioideae	17	100
Duparquetioideae	1	0
Papilionoideae	515	45

### Data comparison 1

Data comparison 1 (Fig. 2) was carried out between the starting list of names (Supplementary Table 1) and the WCVP: Fabaceae checklist^7^. A summary of the checklist data is provided in Table 4, showing the total number of records, the number of accepted species, subspecies, varieties and genera as calculated before and after the checklist was evaluated by the contributors. In total, 2,228 differences in total numbers of accepted taxa and synonyms were recorded in the updated checklist, recognising 772 genera and 22,360 accepted species.

**Table 4:** Summary of the number of accepted genera, species, subspecies and varieties, and synonyms, excluding unplaced taxa.

	2020	2021	Difference
Genera	770	772	2
Species	22441	22360	81
Subspecies	1697	2087	390
Varieties	2540	3047	507
Forms	21	18	3
Synonyms	56824	58069	1245
**Total**			**2228**

These differences in total numbers of accepted taxa and synonyms were recorded in the checklist (Supplementary Table 1) that was circulated for evaluation by contributors (taxon experts) in 2020. The updated version (WCVP: Fabaceae checklist^7^) was published in 2021. The difference in numbers for each category and the total number of differences recorded are listed.

### Data comparison 2

**Fig. 3:**
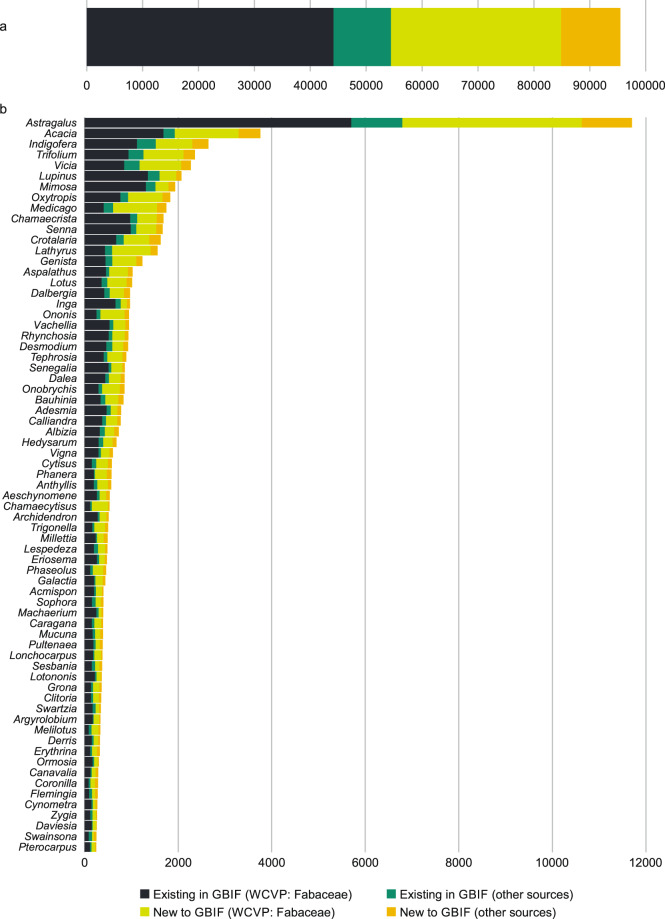
Number of legume names in the GBIF taxonomic backbone before and after updates were introduced through the WCVP: Fabaceae checklist. a. A total of 44,157^10^ names existed in both the GBIF taxonomic backbone and WCVP: Fabaceae while 10,277 names (including fossil names) were not captured in the WCVP: Fabaceae checklist. A total of 30,456^13^ names were added from the WCVP: Fabaceae checklist^7^ into the GBIF taxonomic backbone^10^ while 10,584 names were newly introduced into the GBIF taxonomic backbone through other taxonomic sources after the 2019 snapshot of the GBIF taxonomic backbone was taken. b. Genera with more than 250 newly matched names^10,13^ after the WCVP: Fabaceae checklist was incorporated into the GBIF taxonomic backbone.

In 2019, the Fabaceae component in the initial GBIF taxonomic backbone included 49,656 names (Fig. 3a). In total, 44,157 of these matched names in the WCVP: Fabaceae checklist and a further 10,277 matched names from other data sources (e.g. names coming from the International Legume Database and Information Service (ILDIS)^5^, International Plant Name Index, Plazi). In 2021, after the WCVP: Fabaceae checklist was included in the GBIF taxonomic backbone^10^, a total of 95,474 names were recorded. Of these, 30,456 names were newly introduced through the WCVP: Fabaceae checklist while 10,584 names (including fossil taxa) came from other sources that were not yet captured in the WCVP: Fabaceae checklist. Fossil taxa were not included in the analysis. A total of 69 genera in the GBIF taxonomic backbone had more than 250 new name matches as a result of the WCVP: Fabaceae checklist import (Fig. 3b). Not all of these genera have been curated by the LPWG Taxonomy Working Group yet and the big improvement may be due to continuous work that was carried out by the editor of the WCVP in collaboration with legume taxon experts before the LPWG Taxonomy Working Group was established.

The discrepancies in number of names in these different taxonomic lists point to the multiplicity of taxonomic datasets in the community that are not aligned. To alleviate this problem, the list of names not yet accounted for in the WCVP: Fabaceae checklist can be displayed on the Legume Data Portal and forwarded to the legume community for curation, therefore developing a cycle of data improvement. However, unpublished names coming from observations and herbarium specimens should be omitted. It is anticipated that after a few rounds of expert curation the WCVP: Fabaceae and GBIF taxonomies will converge. At each cycle, a snapshot of GBIF occurrences will be taken and the improvement of the occurrences quantified to measure the value of the expert taxonomic work. By making explicit the logical quantity of records that were improved and ones that had their identifications linked more accurately, the work of taxonomists can be measured, in part, to justify future resource investment in taxonomy.

### Data comparison 3

In the previous two comparisons, improvements within the WCVP: Fabaceae checklist and the GBIF taxonomic backbone were measured. In the third comparison, occurrence data (consisting of observations and specimens) were revised as a result of the updated WCVP: Fabaceae checklist and GBIF taxonomic backbone. This was an automated process and did not include curation of individual occurrences by experts.

**Fig. 4:**
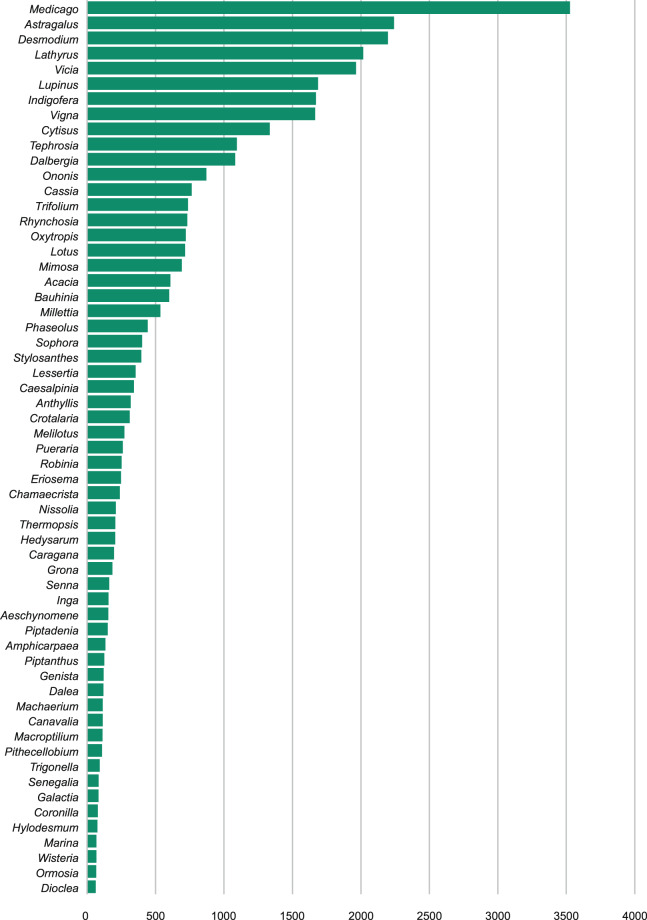
Genera with more than 60 newly matched occurrences after the WCVP: Fabaceae checklist was incorporated into the GBIF taxonomic backbone. These occurrences previously identified to a higher taxonomic rank^11,14^ because of name mismatches at lower ranks.

The GBIF taxonomic backbone was originally constructed from the COL checklist, which was built mainly from data in the International Legume Database and Information Service (ILDIS), World Wide Wattle (http://worldwidewattle.com) and World Plants (https://www.worldplants.de). After the GBIF taxonomic backbone was updated^10^ with the WCVP: Fabaceae checklist^7^, a total of 61,235 occurrences that previously matched to a higher taxonomic level^13^ were improved^11^ and now match to species level or below based on the identification recorded with the occurrence. Of these 42,681 occurrences that matched at the genus level now match to species level or lower while 3,931 occurrences that matched at species level now match to a variety or subspecies level. Family level matches of 14,623 occurrences were improved to match at species rank or lower. This means that previously the verbatim name was not in the GBIF backbone or that there were conflicting names (scientific names with different authorities or concepts) that were now refined and allowed more accurate assignment of the interpreted name at a lower taxonomic level (Figs. 2c, 4). A total of 59 genera were recorded for which 60 or more occurrence records, at various levels, were improved and linked to a lower taxon level as a result of a more comprehensive, curated checklist (Fig. 4).

Even after this taxonomic update, there are still about 250,000 legume occurrences in GBIF that are only identified to a higher taxon level (i.e., are not matched as accurately as they could be). However, more than 56% (about 140,000 occurrences) of these higher rank matches are varieties or subspecies that are moved to species level rank due to missing names, misspellings or missing authorships in the GBIF taxonomic backbone. The other 110,000 occurrences may be improved in their matching at species level through ongoing taxonomic work by the LPWG Taxonomy Working Group. This total includes occurrences published to GBIF that have been assigned only a genus name by the provider, lacking a specific epithet. It will be impossible to improve these matches unless the source data are curated and become more accurate.

By targeting efforts on resolving the missing taxa that result in the identification to genus (or family) level, the LPWG Taxonomy Working Group can have the strongest impact on GBIF occurrences with a result of all (or most) occurrences matching to an accepted species as a LPWG Taxonomy Working Group goal. This contribution of the LPWG Taxonomy Working Group will also provide data publishers with a source of high quality taxonomic data for improving their databases and speeding up reidentification of occurrences. The more comprehensive the GBIF taxonomic backbone becomes through taxon expert curation, the better the matching algorithm will work to assign interpreted names. This will prevent higher taxon level identification of occurrences and increase the accuracy and specificity of data associated with each taxon. The changes in matched occurrences reflect the taxonomic effort by the LPWG Taxonomy Working Group.

Overall, these data comparisons have shown that through collaboration a group of taxonomic experts can efficiently arrive at better quality data for downstream research and applied uses. We illustrate the challenges of conciliating taxa across multiple taxonomic databases and of maintaining synchronised databases that capture conclusions from new taxonomic research. We reiterate the importance of working together to arrive at cleaner taxon databases to aggregate multiple sources of data about biodiversity. This is particularly critical for large families such as Fabaceae, whose size, diversity, economic and ecological importance make it an ideal case study for demonstrating the role of good taxonomy and accompanying databases for accurate research and biodiversity assessments.

### Future work

Collaborative research requires everyone to share the same understanding of a particular taxon concept. These concepts are captured in the community endorsed, consensus checklist that will provide the basis for ongoing collaborative work on phylogenomics, occurrences (species distributions), traits and to contribute towards the Open Tree of Life (www.opentreeoflife.org) through the work planned by these four working groups under the umbrella of the LPWG^4^.

The first goal of the LPWG’s Taxonomy Working Group is to create a single definitive, community-endorsed checklist, which was achieved here, in part. This is an attempt to avoid further and unnecessary duplication of efforts. Many components of the checklist has not yet been verified, e.g. Caesalpinioideae and Papilionoideae subfamilies, which will require repeating the workflow presented in Fig. 1 and expanding the community to include other taxonomists interested to contribute to this ambitious task. Legume taxon experts are invited to contact the author for correspondence to become part of the checklist verification initiative. A list of genera already verified are available in Online-only Table 1. The remaining genera are in various stages of being verified or in some cases, lacking experts, e.g. for some genera in tribes Phaseoleae (pers. comm. Alfonso Delgado Salinas) and Trifolieae (pers. comm. Ana Paula Fortuna). Also, none of the Caesalpinioideae have been verified as the majority of taxon experts are currently working on the next two editions of ‘Advances in Legume Systematics’ (https://phytokeys.pensoft.net/issue/3247/) dealing with the taxonomy of this subfamily. Once taxon changes have been published, an effort will be made to verify and capture these updates in the WCVP: Fabaceae checklist.

Further modifications for genera already checked will have to be maintained in the checklist as new research results become available. Continuous updates to the WCVP: Fabaceae checklist based on queries received from biodiversity data platforms and other users will also help to maintain an accurate checklist. A second goal is therefore to capture names from other online platforms like the GBIF taxonomic backbone, TROPICOS, COL and the World Flora Online Plant List that are unaccounted for in the LPWG: Fabaceae checklist to ensure comprehensiveness, similarly as to what was done for the Cactaceae^16^ and *Dianthus* L.^17^ checklists prepared for the Caryophyllales Network. Workflows may be revised following changes in collaboration agreements among online checklists and biodiversity data portals and with the increasing availability of online tools.

## Usage Notes

The published WCVP: Fabaceae checklist is available through several entry points in open access. These entry points include the Legume Data Portal (https://www.legumedata.org), ChecklistBank (https://www.checklistbank.org/dataset/2304/about) and Zenodo^7^, from which the data can be downloaded, and the World Checklist of Vascular Plants^1,9^ and POWO^9^ (https://powo.science.kew.org) where the data can be visualised.

### The legume checklist usage information

As the legume checklist originates from the WCVP database, a similar format is followed here as recently defined^9^. Each data record has one of the following taxonomic statuses (taxonomicStatus): Accepted, artificial hybrid, illegitimate, incomplete, invalid, misapplied, orthographic variant, synonym or unplaced. Statuses other than accepted and unplaced all point to an accepted name identifier. Records are associated with IPNI and WFO identifiers where these are available.

### Legume data portal

On the Legume Data Portal (https://www.legumedata.org), a Taxonomy tab provides entry points to the latest published checklist through the ‘Browse’ or ‘Advanced search’ options. The portal also provides background information on the checklist, the six subfamilies and static lists of genera within each subfamily using information from the latest published checklist that will be maintained manually. A citation showing the version number is provided at the top of the ‘Browse’ and ‘Advanced search’ pages that should preferably be used when data from the checklist are included in any publication. A link directing the user to the source of the data is listed at the end of the citation. The checklist can be downloaded in a text file format (.txt) or in Word format (traditional style checklist).

### ChecklistBank

The latest version, as well as future versions, of the WCVP: Fabaceae will be available on ChecklistBank. The legume checklist is published within ChecklistBank and provides access to its metadata on the About page. There are also pages for Download, Browse and Search as well as an Import page that displays some statistics of the WCVP: Fabaceae checklist. Searches can be carried out on individual taxa from the Search or Browse pages without having a GBIF account or being logged in, but to download a subset or the entire checklist, the user must have a GBIF account and should be logged into the ChecklistBank portal using the GBIF account credentials. The checklist can be downloaded in the following formats: DwCA, text tree, ColDP, Newick and dot. Alternatively, users can access a URL on the About page that will redirect them to an address that will allow the complete download of the checklist in DwCA without having a GBIF account. The checklist DwCA with World Flora Online identifiers is also available from Zenodo^7^ for download.

The browse function within the ChecklistBank and Legume Data Portal (https://www.legumedata.org) operate in exactly the same way and use the same data source. Here one can search individual records within the dataset or browse through the list of accepted genera, species and infraspecific taxa. Although synonyms are not listed, it is possible to search for a synonym, which will provide the accepted name of the associated synonym. When selecting a name in the list, a taxon summary page will open and display additional information (protologue citation, taxonomic status, associated accepted name or synonyms, basionym and classification information).

### Other biodiversity data portals

Most biodiversity data portals rely on the same pool of taxonomic experts to provide clarification on the use of scientific names. Our goal is to create a primary data source for all legume taxa and to serve these to other online platforms, including the World Flora Online^7,12^. Queries regarding taxa that are not part of our checklist should be communicated to the LPWG Taxonomy Working Group for consideration. In turn, feedback will be provided to help other systems improve their legume taxon backbones.

## Supplementary information

Supplementary Table

## Data Availability

Code used to determine statistics for the WCVP: Fabaceae checklist is available on Github (https://github.com/jhnwllr/The-World-Checklist-of-Vascular-Plants–Fabaceae).

## Online-only Table

**Table-only Table 1:** .

Taxon expert	Subfamily	Genus and author
Atahuachi Burgos, Margoth	Papilionoideae	*Arachis* L.
Balan, Anoop	Detarioideae	*Cynometra* L.
		*Humboldtia* J.Vahl
Bandyopadhyay, Subir	Cercidoideae	*Bauhinia* L.
		*Cheniella* R.Clark & Mackinder
		*Lysiphyllum* (Benth.) de Wit
		*Phanera* Lour.
		*Piliostigma* Hochst.
Barbosa Pinto, Rafael	Detarioideae	*Guibourtia* Benn.
		*Hymenaea* L.
		*Peltogyne* Vogel
Barrett, Russell	Papilionoideae	*Aenictophyton* A.T.Lee
		*Almaleea* Crisp & P.H.Weston
		*Aotus* Sm.
		*Bossiaea* Vent.
		*Chorizema* Labill.
		*Daviesia* Sm.
		*Dillwynia* Sm.
		*Erichsenia* Hemsl.
		*Euchilopsis* F. Muell.
		*Eutaxia* R.Br. ex W.T.Aiton
		*Gastrolobium* R.Br. ex W.T.Aiton
		*Gompholobium* Sm.
		*Goodia* Salisb.
		*Isotropis* Benth.
		*Jacksonia* R.Br. ex Sm.
		*Latrobea* Meisn.
		*Leptosema* Benth.
		*Mirbelia* Sm.
		*Muelleranthus* Hutch.
		*Oxylobium* Andrews
		*Paragoodia* I.Thomps.
		*Phyllota* (DC.) Benth.
		*Platylobium* Sm.
		*Podolobium* R.Br. ex W.T.Aiton
		*Ptychosema* Benth.
		*Pultenaea* Sm.
		*Sphaerolobium* Sm.
		*Stonesiella* Crisp & P.H.Weston
		*Urodon* Turcz.
		*Viminaria* Sm.
Broich, Steven L.	Papilionoideae	*Lathyrus* L.
		*Vicia* L.
Brullo, Savatore	Papilionoideae	*Bituminaria* Heist. ex Fabr.
		*Cullen* Medik.
		*Kartalinia* Brullo, C.Brullo, Cambria, Acar, Salmeri & Giusso
Bruneau, Anne	Cercidoideae	*Bauhinia* Plum. ex L.
		*Cercis* L.
		*Cheniella* R.Clark & Mackinder
		*Lysiphyllum* (Benth.) de Wit
		*Piliostigma* Hochst.
Cardinal-McTeague, Warren	Cercidoideae	*Adenolobus* (Harv. ex Benth. & Hook.f.) Torre & Hillc.
		*Barklya* F.Muell.
		*Bauhinia* L.
		*Brenierea* Humbert
		*Cheniella* R.Clark & Mackinder
		*Gigasiphon* Drake
		*Griffonia* Baill.
		*Lysiphyllum* (Benth.) de Wit
		*Phanera* Lour.
		*Piliostigma* Hochst.
		*Schnella* Raddi
		*Tournaya* A.Schmitz
		*Tylosema* (Schweinf.) Torre & Hillc.
Cardoso, Domingos	Papilionoideae	*Aeschynomene* L.
		*Amphiodon* Huber
		*Behaimia* Griseb.
		*Cristonia* J.H.Ross
		*Cyclolobium* Benth.
		*Haplormosia* Harms
		*Harpalyce* Moç. & Sessé ex DC.
		*Hovea* R.Br. ex W.T.Aiton
		*Hymenolobium* Benth.
		*Lamprolobium* Benth.
		*Limadendron* Meireles & A.M.G.Azevedo
		*Luetzelburgia* Harms
		*Ormosia* Jacks.
		*Petaladenium* Ducke
		*Plagiocarpus* Benth.
		*Poecilanthe* Benth.
		*Sweetia* Spreng.
		*Tabaroa* L.P.Queiroz, G.P.Lewis & M.F.Wojc.
		*Templetonia* R.Br. ex W.T.Aiton
		*Thinicola* J.H.Ross
		*Vatairea* Aubl.
		*Vataireopsis* Ducke
Carlos Andrella, Giovani	Papilionoideae	*Dussia* Krug & Urb. ex Taub.
Carvalho, Catarina Silva de	Papilionoideae	*Dipteryx* Schreb.
		*Monopteryx* Spruce ex Benth.
		*Pterodon* Vogel
		*Taralea* Aubl.
Castro, Isabella C.	Papilionoideae	*Nissolia* Jacq.
		*Trifolium* L.
Cervantes, Angélica	Papilionoideae	*Dalbergia* L.f.
Choo, Le Min	Detarioideae	*Endertia* Steenis & de Wit
		*Intsia* Thouars
		*Leucostegane* Prain
		*Lysidice* Hance
		*Saraca* L.
		*Sindora* Miq.
		*Tamarindus* L.
	Dialioideae	*Dialium* L.
		*Koompassia* Maingay ex Benth.
		*Uittienia* Steenis
Cobra e Monteiro, Thiago	Papilionoideae	*Adesmia* DC.
Compton, James	Papilionoideae	*Adinobotrys* Dunn
		*Austrocallerya* J.Compton & Schrire
		*Callerya* Endl.
		*Nanhaia* J.Compton & Schrire
		*Sarcodum* Lour.
		*Serawaia* J.Compton & Schrire
		*Sigmoidala* J.Compton & Schrire
		*Whitfordiodendron* Elmer
		*Wisteria* Nutt.
		*Wisteriopsis* J.Compton & Schrire
Crameri, Simon	Papilionoideae	*Dalbergia* L.f.
De la Estrella, Manuel	Detarioideae	*Afzelia* Sm.
		*Amherstia* Wall.
		*Annea* Mackinder & Wieringa
		*Anthonotha* P.Beauv.
		*Aphanocalyx* Oliver
		*Augouardia* Pellegr.
		*Baikiaea* Benth.
		*Barnebydendron* J.H.Kirkbr.
		*Berlinia* Sol. ex Hook. f.
		*Bikinia* Wieringa
		*Brandzeia* Baill.
		*Brodriguesia* R.S.Cowan
		*Colophospermum* J.Kirk ex J.Léonard
		*Crudia* Schreb.
		*Cryptosepalum* Benth.
		*Daniellia* Benn.
		*Detarium* Juss.
		*Dicymbe* Spruce ex Benth.
		*Didelotia* Baill.
		*Englerodendron* Harms
		*Eperua* Aubl.
		*Eurypetalum* Harms
		*Gabonius* Wieringa & Mackinder
		*Gilbertiodendron* J.Léonard
		*Gilletiodendron* Vermoesen
		*Goniorrhachis* Taub.
		*Heterostemon* Desf.
		*Hylodendron* Taub.
		*Hymenostegia* (Benth.) Harms
		*Isoberlinia* Craib & Stapf
		*Isomacrolobium* Aubrév. & Pellegr.
		*Julbernardia* Pellegr.
		*Lebruniodendron* J.Léonard
		*Librevillea* Hoyle
		*Loesenera* Harms
		*Michelsonia* Hauman
		*Micklethwaitia* G.P.Lewis & Schrire
		*Neoapaloxylon* Rauschert
		*Neochevalierodendron* J.Léonard
		*Oddoniodendron* De Wild.
		*Oxystigma* Harms
		*Paloue* Aubl.
		*Paramacrolobium* J.Léonard
		*Plagiosiphon* Harms
		*Polystemonanthus* Harms
		*Prioria* Griseb.
		*Pseudomacrolobium* Hauman
		*Schotia* Jacq.
		*Scorodophloeus* Harms
		*Sindoropsis* J.Léonard
		*Stemonocoleus* Harms
		*Talbotiella* Baker f.
		*Tetraberlinia* (Harms) Hauman
		*Zenkerella* Taub.
De Lima, Haroldo C.	Papilionoideae	*Dalbergia* L.f.
De Lima, Haroldo Cavalcante	Papilionoideae	*Dipteryx* Schreb.
		*Hymenolobium* Benth.
		*Poecilanthe* Benth.
Delgado-Salinas, Alfonso	Papilionoideae	*Aeschynomene* L.
Dorado, Oscar	Papilionoideae	*Brongniartia* Kunth
Duan, Lei	Papilionoideae	*Glycyrrhiza* L.
		*Glycyrrhizopsis* Boiss.
Egan, Ashley N.	Papilionoideae	*Pediomelum* Rydb.
Falcão, Marcus	Dialioideae	*Androcalymma* Dwyer
		*Apuleia* Mart.
		*Baudouinia* Baill.
		*Dialium* L.
		*Dicorynia* Benth.
		*Distemonanthus* Benth.
		*Eligmocarpus* Capuron
		*Kalappia* Kosterm.
		*Labichea* Gaudich. ex DC.
		*Martiodendron* Gleason
		*Mendoravia* Capuron
		*Petalostylis* R.Br.
		*Poeppigia* C.Presl
		*Storckiella* Seem.
		*Zenia* Chun
Farruggia, Frank	Papilionoideae	*Sesbania* Adans.
Flores, Andréia S.	Papilionoideae	*Crotalaria* L.
Fortuna Perez, Ana Paula	Papilionoideae	*Ononis* L.
Fortuna, Ana Paula	Papilionoideae	*Zornia* J.F.Gmel.
Fritsch, Peter	Cercidoideae	*Cercis* L.
Giusso del Galdo, Gian Pietro	Papilionoideae	*Bituminaria* Heist. ex Fabr.
		*Cullen* Medik.
		*Kartalinia* Brullo, C.Brullo, Cambria, Acar, Salmeri & Giusso
Goncharov, Mikhail	Papilionoideae	*Airyantha* Brummitt
		*Alhagi* Gagnebin
		*Baphia* Afzel. & Lodd.
		*Baphiastrum* Harms
		*Baphiopsis* Benth. ex Baker
		*Bowringia* Champ. ex Benth.
		*Bracteolaria* Hochst.
		*Calophaca* Fisch. ex DC.
		*Caragana* Fabr.
		*Chesneya* Lindl. ex Endl.
		*Corethrodendron* Fisch. ex Basiner
		*Dalhousiea* Wall. ex Benth.
		*Ebenus* L.
		*Eversmannia* Bunge
		*Greuteria* Amirahm. & Kaz.Osaloo
		*Gueldenstaedtia* Fisch.
		*Halimodendron* Fisch. ex DC.
		*Hedysarum* L.
		*Leucomphalos* Benth. ex Planch.
		*Sartoria* Boiss. & Heldr.
		*Spongiocarpella* Yakovlev & N.Ulziykh.
		*Sulla* Medik.
		*Taverniera* DC.
		*Tibetia* (Ali) H.P.Tsui
Gregório, Bernarda de Souza	Papilionoideae	*Clathrotropis* (Benth.) Harms
		*Panurea* Spruce ex Benth.
		*Spirotropis* Tul.
Kramina, Tatiana	Papilionoideae	*Acmispon* Raf.
		*Lotus* L.
Lachenaud, Olivier	Papilionoideae	*Dalbergia* L.f.
Lana C. Atunes, Lorena	Papilionoideae	*Aeschynomene* L.
Le Roux, Marianne	Papilionoideae	*Crotalaria* L.
Ledis Linares, José	Papilionoideae	*Dalbergia* L.f.
Lewis, Gwilym P.	Unranked	*Neoharmsia* R.Vig.
		*Orphanodendron* Barneby & J.W.Grimes
		*Pericopsis* Thwaites
		*Sakoanala* R.Vig.
Li, Shi-Jin	Papilionoideae	*Dalbergia* L.f.
Mansano, Vidal	Dialioideae	*Dialium* L.
Mashego, Kagiso	Papilionoideae	*Cullen* Medik.
Mattapha, Sawai	Papilionoideae	*Afgekia* Craib
		*Endosamara* R.Geesink
		*Kanburia* J.Compton, Mattapha, Sirich. & Schrire
		*Padbruggea* Miq.
Montenegro Valls, Jose Francisco	Papilionoideae	*Arachis* L.
Murphy, Bruce	Detarioideae	*Macrolobium* Schreb.
Ohashi, Hiroyoshi	Papilionoideae	*Akschindlium* H.Ohashi
Ohashi, Kazuaki	Papilionoideae	*Akschindlium* H.Ohashi
Pandey, Arun	Papilionoideae	*Crotalaria* L.
Pennington, R. Toby	Papilionoideae	*Andira* Lam.
Phillipson, Pete	Papilionoideae	*Dalbergia* L.f.
Povydysh, Maria	Papilionoideae	*Alexa* Moq.
		*Angylocalyx* Taub.
		*Castanospermum* A.Cunn. ex Hook.
		*Uleanthus* Harms
		*Xanthocercis* Baill.
Rakotonirina, Nivohenintsoa	Papilionoideae	*Dalbergia* L.f.
Ramos, Gustavo	Papilionoideae	*Aldina* Endl.
Ranzato Filardi, Fabiana	Papilionoideae	*Machaerium* Pers.
Sanjappa, Munivenkatappa	Papilionoideae	*Aeschynomene* L.
Santos-Guerra, Arnoldo	Papilionoideae	*Genista* L.
		*Lotus* L.
		*Rivasgodaya* Esteve
Sartori, Ângela Lúcia Bagnatori	Papilionoideae	*Cordyla* Lour.
		*Mildbraediodendron* Harms
		*Myrocarpus* Allemão
		*Myrospermum* Jacq.
		*Myroxylon* L.f.
Schley, Rowan	Detarioideae	*Brownea* Jacq.
		*Browneopsis* Huber
		*Ecuadendron* D.A.Neill
Schrire, Brian	Papilionoideae	*Indigofera* L.
		*Nanhaia* J.Compton & Schrire
		*Serawaia* J.Compton & Schrire
Schütz Rodrigues, Rodrigo	Papilionoideae	*Bowdichia* Kunth
		*Camoensia* Welw. ex Benth.
		*Diplotropis* Benth.
		*Guianodendron* Schutz Rodrigues & A.M.G.Azevedo
		*Leptolobium* Vogel
		*Staminodianthus* D.B.O.S.Cardoso, H.C.Lima & L.P.Queiroz
Seijo, Guillermo	Papilionoideae	*Arachis* L.
Seleme, Elidiene Priscila	Papilionoideae	*Amburana* Schwacke & Taub.
Simpson, Charles E.	Papilionoideae	*Arachis* L.
Sirichamorn, Yotsawate	Papilionoideae	*Afgekia* Craib
		*Endosamara* R.Geesink
		*Kanburia* J.Compton, Mattapha, Sirich. & Schrire
		*Padbruggea* Miq.
Soares Gissi, Danilo	Papilionoideae	*Melilotus* Mill.
		*Parochetus* Buch.-Ham. ex D.Don
		*Trifolium* L.
Sokoloff, Dmitry	Papilionoideae	*Acmispon* Raf.
		*Anthyllis* L.
		*Antopetitia* A.Rich.
		*Coronilla* L.
		*Cytisopsis* Jaub. & Spach
		*Dorycnium* Mill.
		*Dorycnopsis* Boiss.
		*Hammatolobium* Fenzl
		*Hippocrepis* L.
		*Hosackia* Douglas ex Lindl.
		*Kebirita* Kramina & D.D.Sokoloff
		*Lotus* L.
		*Ornithopus* L.
		*Ottleya* D.D.Sokoloff
		*Podolotus* Royle
		*Pseudolotus* Rech.f.
		*Scorpiurus* L.
		*Securigera* DC.
		*Tripodion* Medik.
Stirton, Charles H.	Papilionoideae	*Cullen* Medik.
		*Hoita* Rydb.
		*Orbexilum* Raf.
		*Otholobium* C.H.Stirt.
		*Psoralea* L.
		*Psoralidium* Rydb.
		*Rupertia* J.W.Grimes
Subramaniam, Shweta	Papilionoideae	*Crotalaria* L.
Thulin, Mats	Papilionoideae	*Chapmannia* Torr. & A. Gray
Van der Burgt, Xander	Papilionoideae	*Dalbergia* L.f.
Wilding, Nicholas	Papilionoideae	*Dalbergia* L.f.
Wojciechowski, Martin	Papilionoideae	*Cladrastis* Raf.
		*Pickeringia* Nutt. ex Torr. & A.Gray
		*Platyosprion* Maxim.
		*Sesbania* Adans.
		*Styphnolobium* Schott
